# Sex differences in the response to treatment of attention deficit hyperactivity disorder

**DOI:** 10.1007/s00210-025-04716-5

**Published:** 2025-10-16

**Authors:** Elisa D. Müller, Anke C. Fender

**Affiliations:** 1 Practice for Psychological and Behavioural Psychotherapy, Lünen, Germany; 2 Institute for Pharmacology, Medical Faculty, University Duisburg-Essen, Hufelandstr 55, 45147 Essen, Germany

**Keywords:** Sex, Attention deficit disorder with hyperactivity (ADHD), Central stimulants, Psychostimulants, Methylphenidate, Amphetamines

## Abstract

Attention deficit hyperactivity disorder (ADHD) impacts markedly on juvenile development and daily function of young and adult patients. Global prevalence and drug prescription rates are consistently on the rise, particularly in young women. Personal experience in daily psychotherapy practice supports sex differences in how the disorder manifests and how the patients respond to treatment. We review sex differences in therapeutic and adverse responses to the cornerstone ADHD pharmacotherapies methylphenodate and amphetamines, under consideration of preclinical mechanistic insights, clinical studies, and relevance for real-life psychotherapy practice. Overall, many gaps in knowledge remain regarding sex differences on all levels of ADHD disease manifestation, diagnosis, and therapy, and the underlying mechanistic basis. Certain treatment strategies may be more or less appropriate, safe, and effective in specific patients, an aspect which warrants further attention in future guideline reforms.

Attention deficit hyperactivity disorder (ADHD) features a persistent state of inattention, hyperactivity and lack of impulse control, which impacts markedly on juvenile development and daily function of the afflicted young and adult patients. Global prevalence and drug prescription rates are consistently on the rise (Board et al. 2020; Bruno et al. 2023; Sørensen et al. 2023; Cho et al. 2024), particularly in young women. Regarding sex-specific differences in the context of ADHD, personal experience in daily psychotherapy practice supports differences in how the disorder manifests and how the patients respond to treatment.

During preparation of this manuscript, two excellent narrative reviews were published. Amiri et al. (2025) summarize across 35 studies sex-specific disparities in ADHD diagnosis and medication efficacy. The authors highlight that the continued underrepresentation of females in ADHD research skews current treatment guidelines, and does not consider biological, sociocultural and symptomatic differences that may need more targeted medication strategies. Rapoport and Groenman (2025) applied a theoretical framework to identify five critical gaps in knowledge related to sex-determined outcome of psychostimulant treatment: (i) overall sex-differences in psychostimulant treatment efficacy, (ii) adverse drug reactions in females versus males across age-groups, (iii) impact of menstrual cycle and reproductive life transitions on treatment efficacy, (iv) sex-differences in treatment adherence, and (v) sex-dependent influence of ADHD-associated stigma and camouflaging.

In the following, we also touch on these topics and specifically discuss also pre-clinical studies that provide candidate mechanistic explanations for clinical observations. We provide an overview of how sex differences manifest in ADHD treatment, under consideration of scientific data on the molecular pathways, clinical studies and real-life accounts from psychotherapy practice.

## Sex differences in ADHD prevalence, diagnosis and manifestation

Male children and adolescents are generally more frequently diagnosed with ADHD than female counterparts. Accordingly, ADHD was historically seen as a “young male issue” (Concannon and Schechter 1981). While this could reflect a true difference in prevalence, it may also indicate a relative under-diagnosis and referral of afflicted girls and women. ADHD manifests somewhat differently between male and female subjects (Robison et al. 2008), as also exemplified by the two cases, Henry and Emilia, described in Box 1. The case studies are modified and collated from multiple real cases to best recapture the relevant patient profile.

**Box 1:**
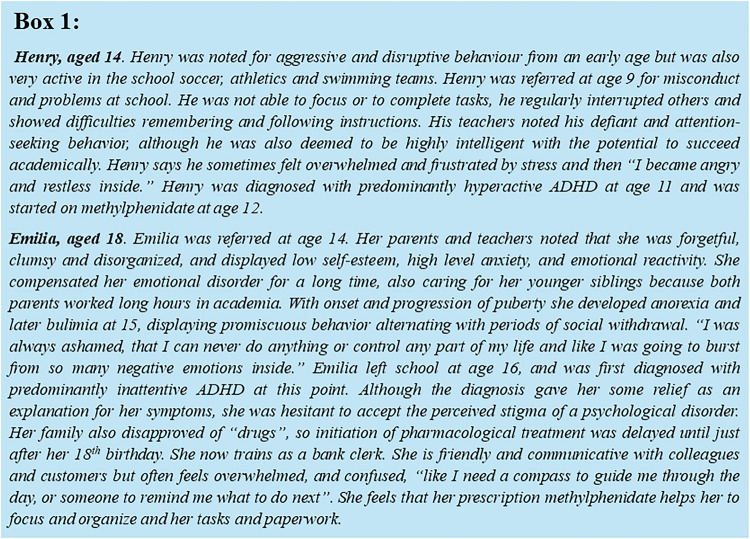
Cases of Henry and Emilia

As represented by Henry, males affected by ADHD show a symptom cluster encompassing hyperactivity, aggression and impulsive behavior (Gualtieri et al. 1985). Greater impulsivity, disrupted appetite hormone levels and impaired cognitive function were recently linked with low-grade inflammation, as part of a newly proposed immune-metabolic ADHD subtype that is more frequent in male adolescents than female counterparts (Chen et al. 2025). The symptoms of typically male ADHD often present from early childhood, as in Henry’s case. His inability to vent stress and restlessness despite regularly sports activity impacted notably on his early academic success, and led to his timely referral and therapy.

Emilia by contrast personifies a typical female ADHD patient, tending to display inattention and other less overt symptoms, compared to the hyperactive-impulsive behavior frequently manifested in males. Emilia also displays the high-risk sexual behavior of youngsters with ADHD, which in females, more so than in males, manifests as younger onset of sexual activities, higher number of partners and less use of barrier protection (Young et al. 2023). ADHD was previously reported to go in hand with marked monetary and sexual delay discounting, with more severe ADHD symptoms being associated with greater preference for immediate unprotected sex. In this regard, no differences were noted between males and females (Berry et al. 2021). The less disruptive nature of female ADHD may make it less likely to be recognized; moreover, the associated difficulties could, as in Emilia’s case, be compensated for by a relative pro-social behavior in girls and women. Also, in females, the symptoms of ADHD could be masked by a different spectrum of co-existent disorders such as depression, anxiety, eating disorders or sleep disorders, and hormonal fluctuations (Quinn 2005; Rucklidge 2010; Skogli et al. 2013; Martin 2024). As with Emilia, the compensatory nature of female ADHD often delays diagnosis and medical treatment, compared to boys. In the case of Emilia, her own and her family’s view of ADHD and ADHD drug therapy, and the perceived associated stigma, further delayed initiation of stimulant pharmacotherapy. The characteristics and implications of stigma are beyond the scope of this review but have been well-addressed previously (Pescosolido et al. 2008; Taylor and Antshel 2021; Pavlova and Berkers 2022; Cripe et al. 2025). Although in adulthood, sex differences in prescription patterns become less significant (Klora et al. 2016; Kok et al. 2020), a critical consideration in this context however is that delayed initiation of pharmacological treatment in female adolescent ADHD patients may have a long-term impact on academic progress. Specifically for mathematical achievements, a greater decline was noted in girls with ADHD compared with boys when pharmacotherapy was commenced later rather than early after diagnosis (Zoëga et al. 2012). Finally, female sex as well as an emergence of ADHD in early adulthood plus a delayed treatment onset (Dalsgaard et al. 2014; Elkins et al. 2020) have all been associated with an increased vulnerability to substance and alcohol abuse in later life. The sequelae of delayed treatment likely extend far beyond mathematical achievement and substance abuse, however. Potentially, undiagnosed and/or untreated women with ADHD could be more likely to receive a false diagnosis, for example of borderline personality disorder, and to be at higher risk for other mental health disorders, unplanned pregnancy or sexually-transmitted diseases.

## Sex specific differences in major pathways underlying ADHD

Restoration of improper neurotransmitter signaling is a cornerstone of pharmacological ADHD therapy. The main misregulated transmitters in this context are dopamine and noradrenaline, which direct cognitive alertness and vigilant concentration respectively. Preclinical evidence supports sex-specific differences in both neurotransmitter systems, which may to a large extent, but not fully, be attributed to the influence of sex hormones.

Recent reviews comprehensively outline the current understanding—predominantly gleaned from preclinical observations—of male versus female dopaminergic (Williams et al. 2021; Zachry et al. 2021; Lewitus et al. 2024) and noradrenergic (Joshi and Chandler 2020) platforms, and how these may be influenced by sex hormones. The clinical verification is still largely missing however. Female rodents display a higher noradrenaline efflux in the prefontal cortex than males (Horvat et al. 2024), as well as higher density and dendritic complexity of noradrenergic neurons (Pinos et al. 2001; Bangasser et al. 2011; Mulvey et al. 2018). A whole-brain functional map of distinct cellular noradrenaline systems (Sulkes Cuevas et al. 2023) identified a sexually dimorphic regulation of locus coeruleus (LC)-derived noradrenaline and the respective neuronal populations. The novel observation by Sulkes Cuevas et al. was that sex differences are also evident specifically in hippocampal connections to LC noradrengergic neurones projecting to the amygdala, with this specific circuit being more prominent in female mice.

A sex-specific regulatory circuit involving dopamine D2 receptors and the dopamine clearance transporter DAT was also recently uncovered to differentially modulate dopamine signaling and behavioral outcome in male versus female mice (Mayer et al. 2024). The elegant experimental approach utilized genetic knock-in of the DAT Val559 variant, which has been identified in boys with ADHD and leads to a dysregulated dopamine efflux and tonic activation of dopamine D2 receptors. How this relates to the clinical evolution of ADHD and drug treatment responses in males and females warrants further study. A brain imaging study in healthy human females, without ADHD diagnosis, assessed the interplay between oral contraceptive (OC) use and dopaminergic signals. OC users displayed increased capacity for dopamine synthesis and a greater cognitive flexibility, although D2/D3 receptor binding was comparable (Taylor et al. 2023). Notably, the differences observed between OC users and non-users was greater than the difference noted between the pooled female cohort versus males, clearly showing that comparisons made on the basis of sex alone may mask dynamic changes in response to hormonal status. This study exemplifies that hormonal status either by natural cycle or OC use, can modulate dopaminergic neurotransmission. This should be kept in mind when interpreting data suggesting the presence—or absence—of sex-differences related to ADHD pathophysiology and treatment outcome. Overall, estradiol appears to augment dopamine levels in the dorsal striatum, while dynamically regulating the expression and activity of the dopamine clearance transporter DAT as well as the dopamine D2 auto-receptor expression and affinity. Male rodents generally display a lower baseline D2 receptor density compared to females, for example (Izquierdo et al. 2016).

## Frontline drugs for the treatment of ADHD

ADHD is highly contextual, frequently occurring in conjunction with other mood and functional disorders such as depression or anxiety, eating disorders, or autism (Azim et al. 2025, Brancati et al. 2025, Juli et al., 2025, Torres et al. 2025, Wong et al. 2025). A multimodal management is therefore required, encompassing both psychotherapy, such as cognitive behavioral therapy, and pharmacotherapy. The field of neurostimulation for ADHD is progressing rapidly (Baweja et al. 2024; Ostinelli et al. 2025; Pasca et al. 2025), but this approach is outside the scope of this review. The pillar of ADHD pharmacotherapy is the psychostimulants for symptomatic improvement. Most widely used are methylphendiate and amphetamines, with atomoxetin and guanfacine as second-line therapy. Alternatives are also provided by bupropion, clonidine, and modafinil (Cortese et al. 2018; Ostinelli et al. 2025). In the following, we will briefly introduce the most frequently prescribed drugs for ADHD treatment, specifically methylphenidate for which most evidence exists, as well as the amphetamines and atomoxetine, for which the data is more sparse. We highlight sex-differences in the clinical context where available. In the subsequent section, we will outline preclinical studies that provided deeper insight into sex-differences at the molecular and cellular level. A simplified schematic summary of the molecular mechanisms of action of methylphenidate and amphetamines is provided in Box 3.

### Methylphenidate

The preferred first-choice medication for short-term treatment of ADHD is methylphenidate (e.g., Medikinet®, Ritalin®, Concerta®), a central nervous system stimulant that is available as an immediate-release and as a retard preparation (Cortese et al. 2018). Its precise mechanism of action remains incompletely elucidated but is largely attributed to inhibition of dopamine and noradrenaline uptake transporters in presynaptic neurones (Vedrenne-Gutiérrez et al. 2024). This increases the levels of both neurotransmitters in the synaptic cleft, as a consequence of greater post-synaptic transmission, attention, concentration and cognitive performance are improved (Verghese et al. 2024). Additionally, methylphenidate can—unlike other psychostimulants—increase neuronal firing rate (Viggiano et al. 2004) and is also a weak agonist at the serotoninergic 5HT1A receptor, thereby boosting levels of dopamine (Markowitz et al. 2009).

Methylphenidate is rapidly and near-completely resorbed upon oral intake and undergoes prominent first-pass metabolism by carboxylesterase 1 (CES1), generating the pharmacodynamically inactive ritalinic acid (Markowitz and Melchert 2022). A moderately (approximately 20%) higher CES1 expression in females (Shi et al. 2016) is reflected by respective differences in methylphendiate exposure (Xiao et al. 2022). Overall, methylphenidate is generally well-tolerated, and older data suggest that clinical responses are largely independent of socioeconomic status, lifetime history of psychiatric comorbidity, and sex, at least over short-term use (Pelham et al. 1989; Spencer et al. 2005; Mikami et al. 2009). A recent study from the Children’s Hospital of Fudan University, China, provides data on 533 children treated with methylphenidate (465 boys and 68 girls), of which the majority were diagnosed with either inattentive or inattentive/hyperactive/impulsive ADHD (Zhang et al. 2024). Parents reported marked improvement in the clinical symptoms of ADHD in 396/465 (85%) of boys and in 55/58 (81%) of girls treated with methylphenidate. Adverse events were experienced by 229/465 (50%) of boys and 26/68 (38.2%), most commonly loss of appetite leading to weight-loss, as well as sleep disturbances and to a lesser extent psychiatric problems such as emotional instability, irritability, aggression, and mood disorders. The causal contribution of methylphenidate, distinct from the underlying ADHD, was not clear from the data provided. This apparent sex difference in reported adverse events was not statistically significant, perhaps because of the low number of girls included. Other side-effects of methylphendiate can include tics, dizziness, abdominal pain, headaches, nausea, vomiting, transient transaminase elevation and chest tightness, palpitations, or tachycardia (Verghese et al. 2024; Zhang et al. 2024). Pharmacovigilance data has also associated methylphendiate use in children with growth retardation and lower bone density, dyskinesia and later life risk of hypertension and myocardial infarction (Wei et al. 2023). A higher propensity was noted in male compared to female children and adults. However, these functional relationships are highly context-dependent and the causal links are not yet clear. The recently published attention deficit hyperactivity disorder drugs use chronic effects (ADDUCE) study demonstrated a generally safe profile of methylphenidate over a longer term (24 months) use in children, with no notable adverse impact on growth, although higher pulse rate and blood pressure were documented in methylphendiate users. ADDUCE enrolled approximately 82% and 84% boys in the ADHD with and without methylphenidate groups respectively, while the control group encompassed 45% males; the comprehensive analysis however did not specifically assess sex differences (Man et al. 2023), so that longer-term clinical data remains a critical gap in knowledge.

Sex differences appear to exist in terms of the methylphenidate-activated dopaminergic reward system. Brain imaging of male and female subjects uncovered greater dopamine release in the ventral striatum of females in response to oral or intravenous methylphenidate, associated with increased reporting of a subjective drug-effect experience (Manza et al. 2022). No differences between the sexes were noted in dorsal striatium or plasma levels of dopamine. These observations imply that females may show enhanced sensitivity to methylphenidate in terms of the dopaminergic reward system and may in a wider sense be more vulnerable to substance abuse. Intruigingly, the sex differences in subjective methylphenidate responsivenes are of a temporal nature, with female adolescents reporting a stronger subjective drug effect 90 min after dosing compared to males, but a lower delayed response after 12 h (Sonuga-Barke et al. 2007). This time-dependent patterns in the subjective drug response appears consistent across different methylphenidate formulations, suggesting that male and female patients may require differentially titrated extended release-once daily formulations. Some preliminary work on individualized dose optimization requires further translation into the clinical context however (Huss et al. 2017). An additional benefit of placebo-controlled methylphenidate titration may be the timely identification of placebo-responders and non-responders, to ultimately reduce overtreatment and improve symptom control in those benefiting from methylphenidate (Vertessen et al. 2024).

The dopaminergic influence also extends to reward values of food, modulating macronutrient preferences with an overall reduction in fat and cardbohydrate intake (Verghese et al. 2024). The normal dosages of methylphenidate used in ADHD therapy are under the threshold for activation of the reward system, but overdosing drive may drive expression of the transcriptional activator ΔFosB in striatal neurons (Kim et al. 2009). This has been linked with reward and motivational and can promote anorexia and cachexia (Olausson et al. 2006; Ruud and Blomqvist 2007). In keeping with the stronger and earlier emotional response to methylphenidate, together with a more sustained subjective drug effect in females compared to males (Davis et al. 2016), obese women tend to report a greater reduction in food craving and appetite than while on methylphenidate (Davis et al. 2012; Vedrenne-Gutiérrez et al. 2024). It is as yet unclear if males are in fact comparatively resistant to the anorectic effects of methylphenidate, or if the available studies are underpowered to detect a robust response. In light of an increasing prevalence of obesity and binge-eating disorders, distinct dampening responses to stimulants in women, and the underlying pathways, warrant further consideration in research and practice.

### Amphetamines

Amphetamines are provided as mixed amphetamine salts (e.g., Adderall®) or single preparations of amphetamine sulfate (e.g., Evekeo®), dexamphetamine sulfate (e.g., Dexedrine®, Attendin®), or the prodrug lisdexamfetamine dimesylate (e.g., Vivanse®, Elvanse®). Data on sex differences in the response to these amphetamines is quite limited and not consistent. A double-blind cross-over laboratory classroom study in pediatric ADHD patients aged 6–12 years assessed sex as a potential moderator of responsiveness to amphetamine treatment. Males and females had comparable baseline ADHD rating scores at study entry and at the end of the open-label dose-optimization period. Both also responded well to treatment with comparable onset and duration of effect (Childress et al. 2019). Potentially, sex differences in amphetamine responses emerge during adolescence and adulthood, when hormonal regulators come into play. One pharmacokinetic study of lisdexamfetamine dimesylate in healthy older adults showed that the d-amphetamine Cmax—the peak plasma concentration of the active metabolite reached after oral application of the prodrug—is consistently, albeit marginally, higher in women while the elimination half-life is greater in men. Overall, however, d-amphetamine parameters were comparable between the sexes (Ermer et al. 2013).

Our case study of a young female patient off oral contraception (Box 2) exemplifies how the menstrual cycle can affect response to ADHD treatment. Janine is also a fictional patient whose symptoms recapitulate the typical profile of young women in psychotherapy practice. Janine does not feel adequately supported by her amphetamine medication in the days leading up to menstruation, indicating that drug bioavailability and/or efficacy may be regulated by hormonal variations. This fits with a dynamic regulation of hepatic CYP450 metabolizing enzymes by estrogen and progesterone (Choi et al. 2013; Konstandi et al. 2020) and the observation that higher CYP2D6 metabolizing capacity for example significantly lowers the odds of symptom improvement in children and adolescents treated with amphetamines (Gerlach et al. 2024). That estrous cycle hormones dynamically influence responsiveness to a single oral amphetamine dose was verified in healthy, normally cycling women stratified by cycle phase, and compared to healthy men. Females reported a subjectively greater self-reported stimulation during their follicular versus luteal phase, which correlated positively with pre-drug salivary estradiol and negatively with salivary progesterone. The amphetamine response during the female luteal phase was also lower than that reported by males (White et al. 2002). Therefore, higher estrogen and lower progesterone levels could be associated with a greater subjective stimulation in response to amphetamines in women. In support of this, a later study identified sex-specific quantitative and regional difference in the dopamine release response to oral amphetamine. Healthy females showed an overall greater amphetamine-triggered dopamine release than males, and this difference was also noted in the right globus pallidus and right inferior frontal gyrus as well as the temporal and parietal cortices. Males by contrast showed greater dopamine release in the dorsal striatum upon amphetamine challenge (Riccardi et al. 2006). Menstrual cycle or hormonal contraception should ideally be considered as confounders in studies examining treatment responses, in order to capture sex-based differences.

**Box 2:**
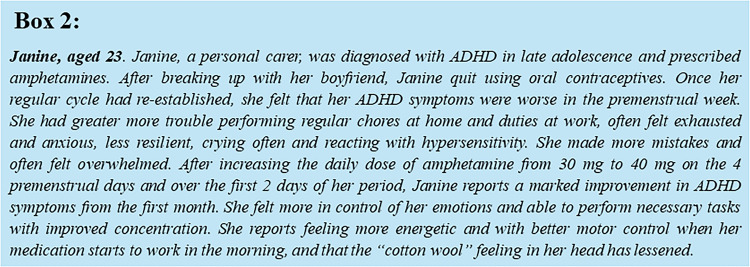
Case of Janine

Hormonally regulated stimulant responses could also lead to different degrees of sensation-seeking and cognition in males and females. In line with this, amphetamine has been reported to acutely heighten the relationship between a sweet taste sensation and reward responses in women but not in men (Weafer et al. 2017). This might imply a vulnerability to substance abuse that might need to be considered in women prescribed amphetamines for ADHD. However, the perceived sex difference in amphetamine-triggered dopamine release not ubiquitously observed. A more recent brain scanning study detected no robust differences between male and female subjects, or between women on versus off birth-control. There was also no correlation between dopamine release and plasma estradiol (Smith et al. 2019). The relatively small sample number (under 20) may have been inadequate to detect subtle differences, but whether these are in any case sufficient to notably impact on the success of ADHD therapy warrants further study. Overall, clinical responses to racemic amphetamine sulfate and lisdexamfetamine in children appear to be comparable between the sexes (Mikami et al. 2009; Turgay et al. 2010; Childress et al. 2019) although there is some indication that male children may more frequently meet the definition of “responder” to lisdexamfetamine sulfate than girls (McGough et al. 2012).

### Atomoxetine and guanfacidine

There is a dearth of data on these agents in terms of sex-specific differences, and what little there is, relates largely to adverse effects. The therapy response rates for boys and girls with ADHD are comparable between the sexes and largely comparable across age and body weights (Naya et al. 2021). Efficacy of atomoxetine lies around 63% and 67% in boys and girls respectively. Adverse effect rates are also comparable at 56 and 58% respectively (Zhang et al. 2024) but lead more frequently to discontinuation in females (Naya et al. 2021). A small study in 41 patients (24 women) suggested that women may be prone to QT prolongation correlating with atomoxetine dose, while cardiotoxicity and dosage relationship are absent in males (Suzuki et al. 2017). This stark sex-difference was not found however in a large-scale pharmacovigilance analysis based on the FDA Adverse Event Reporting System (FAERS) (Wei et al. 2023), in which QT prolongation was more prominent in atomoxetine-treated males up to18 years of age. The vulnerability to cardiac rhythm disturbances—regardless of sex—should be considered when a risk of QT prolongation exists or may be precipitated by drug interactions. For guanfacidine, serious adverse events appear to be uncommon; the most frequent treatment-emergent adverse events include somnolence, insomnia, headache, blurred vision, and altered mood. Severity is largely mild-moderate and more common with high-dose treatment. Younger and lower-weight children may be more prone due to higher plasma concentrations and related pharmacokinetic parameters (Boellner et al. 2007). Sex-differences have not been specifically reported.

## Insights from preclinical models into sex-specific responses to ADHD medication

### Methylphenidate

Comparison of male and female rats has revealed distinct responses to methylphenidate in various developmental stages and stress contexts, although by and large clinical verification is still required. As seen in the clinical context, a stronger sensitivity to methylphenidate prevails in female over male test animals. One explanation is a slower brain clearance of especially the active d-enantiomer of the drug and a consistently higher and more sustained overall methylphenidate concentrations in whole brains of female versus male rats after methylphenidate injection (Bentley et al. 2015). Another cause may be a higher baseline and methylphenidate-stimulated noradrenaline efflux in the prefrontal cortex of female versus male rats (Horvat et al. 2024). This translated into an enhanced active waking and arousal of female rats subjected to traumatic brain injury, which may be of clinical relevance given that ADHD is linked with an increased rate of accidents with concussion or head trauma (Wolff et al. 2019; Brunkhorst-Kanaan et al. 2021). In juveline Sprague–Dawley rats, oral methylphenidate over 3 months has been reported to evoke stronger locomotor activation, hyperactivity and anxiolytic and antidepressant actions in females compared to males (Robison et al. 2017; Martin et al. 2018). To offset the higher locomotor activity, females were noted to increase food intake, while in males, fluid intake was reduced (Robison et al. 2017), although reductions in body weight appear to be similar in males and females (Martin et al. 2018). Adolescent rats of both sexes given methylphenidate by intraperitoneal injection over postnatal day (pnd) 33–49 showed an acute locomotor suppression at low doses, while higher doses evoked locomotor sensitization and behavioral activation especially in females compared to males. This was associated with a notable suppression of brain-derived neurotrophic factor in the striatum of female rats but a marked increase in males (Brown et al. 2012).

In a recent report (Jung et al. 2023), spontaneously hypertensive rats (SHR), a strain that displays ADHD-typical characteristics, were subjected to chronic unpredictable mild stress on pnd 25–32. The juvenile stress conditioning consisted of a constellation of 13 stressors performed 3 × per day, including restraint, food deprivation, forced swim, light on and cage rotation, repositioning or tilt. Male rats developed more stress-triggered hyperactivity than females, which in turn displayed a greater degree of memory impairment. Methylphenidate applied in the post-stress phase of pnd 33–39 led to a stronger improvement of ADHD symptoms in male versus female rats. Transcriptional assessment of the dopaminergic system revealed that genes related to dopamine turnover and clearance, including the dopamine transporter DAT, was suppressed to a greater extent in methylphenidate-treated males compared to females.

The exposure to methylphenidate during juvenile neuro-development may also result in a sex-specific long-term impact on drug-dependent behavior and probabilistic decision-making in adulthood. In SHR conditioned with methylphenidate during adolescence (pnd 29–57), later re-exposure led to a greater drug-conditioned place preference in female SHR; locomotor stimulating effects of the later methylphenidate challenges did not differ however (Yates et al. 2023). Sex differences have also been reported in terms of an enduring effect of adolescent methylphenidate exposure on learning flexibility and risk-preferences into adulthood, in part by remodeling dopamine receptor expression (Izquierdo et al. 2016; Kelly et al. 2022).

Male and female Long–Evans rats exposed to methylphenidate for a limited time during adolescence showed no overt differences in adult reversal learning in the elevated plus maze. Discrimination learning was somewhat slower in females, but methylphenidate pre-exposure did not affect this. The authors suggested that the earlier maturity in females may have determined the extent and type of outcome, as well as baseline difference in anxiety and explorative behavior. Female rats showed less readiness to engage in open versus closed arm entries, which may have influenced the rate of discrimination learning. The time spent in open and closed arms was however not different between the sexes, nor did methylphenidate pre-exposure have an effect on this. Of note, striatal D2, but not D1, was selectively upregulated in adult female rats treated with methylphenidate in adolescence, with no such treatment effect in males. Differential analysis of dopaminergic pathways in striatal sub-regions as well as in regions more closely linked with behavioral learning might unmask sex-dependent and treatment responses not evident in the analysis reported here. In a similar study, Long–Evans rats were exposed to methylphendiate in pre-pubescence (pnd 10–29) and were in adulthood (pnd 60 onwards) trained for low- and high-risk decision-making models: one piece of sugar on a fixed schedule versus three sugars on a variable schedule. Females tended to the low-risk choice more frequently than males, who more often preferred the high-risk option. In this study, no anxiety- and locomotor assessment in an open-field test showed any obvious sex-specific differences.

It must be noted that the Long–Evans outbred rat strain used in these two studies is not a suitable translational model to study ADHD, and observations here rather serve to indicate the long-term impact of an isolated early methylphenidate exposure. The same may also be true for the Wistar and Sprague–Dawley strains, but insights gained from such studies may yet have translational value in the context of sex-dependent methylphenidate treatment outcome. One recent study assessed in detail how methylphenidate differentially affects the biochemistry and bioenergetics of the developing brain in juvenile Wistar rats (Rieder et al. 2024). An estrogenic component and different estrogen-controlled processes at baseline may buffer or differentially modulate responses to methylphenidate applied during juvenile neurodevelopment. In male Wistar rats, the prefrontal cortex displayed higher activity of the respiratory chain complex I at baseline compared to females. Methylphenidate reduced activity of complex I and to a modest extent also of complex IV, and increased levels of the mitochondrial fission marker dynamin-related protein 1 (Drp1). Mitofusin-2 (Mfn2) was by contrast downregulated, while the mitochondrial transcription and packaging factor Tfam was upregulated. Altogether, this constellation of changes would favor together mitochondrial redox stress, biogenesis and reorganization.

In female rats, the estrogenic milieu favored adaptive redox stress (Rieder et al. 2024), reflecting an earlier study (Brown et al. 2012) that showed methylphenidate applied to adolescent rats depressed the antioxidant defenses superoxide dismutase (SOD) and catalase in the cortex of both sexes, but in males additionally depleted glutathione and glutathione peroxidase. Bioenergetic responses to methylphenidate also differed in females compared to males, with notably increased activities of complex III and IV and a significant reduction in ATP levels in the prefontal cortex. These differences were partially attributed to sex-dependent differences in the baseline and methylphenidate-regulated abundance of the mitochondrial regulatory proteins NRF1 and Parkin. Female rats expressed higher baseline NRF1 than males, possibly through estradiol-derived regulation (Zhang et al. 2017; Klinge 2020), while Parkin levels were largely comparable between the sexes. In response to methylphenidate, NRF1 expression was subdued in females but negligibly affected in males. Parkin was upregulated solely in female rats upon methylphenidate, reflecting an estradiol-dependent regulatory and signaling interplay(Spratt et al. 2014). Estrogen does not however explain all sex-based differences in methylphenidate responses. In adolescent Wistar rats treated for up to 7 days, processes related to attention, filtering and processing of stimuli were impaired both acutely (within 24 h) and in a sustained manner over the 7-day exposure in males only. Females remained largely unaffected in the model of prepulse inhibition, regardless of estrous cycle phase (Montiel-Herrera et al. 2024).

Finally, off-target adverse actions of methylphenidate also appear to differ between the sexes. A notable example is a detrimental impact on bone quality reported in young, 4-week old Sprague–Dawley rats (Uddin et al. 2018). Females were noted to modestly lose bone mineralization, while males showed a striking loss of biomechanical bone integrity, related to a higher osteoclast differentiation and bone resorption activity. The potential for a heightened risk of bone fractures and early osteoporosis specifically in males warrants further attention.

### Amphetamines and atomoxetine

The preclinical data on these ADHD medications is more limited compared to methylphenidate, although some studies in rats have highlighted distinct responses males and females (Box 3). Female rats treated with amphetamines for example show a greater rotation activity (Brass and Glick 1981) and a lower propensity for subsequent ethanol intake (Ruiz et al. 2018) than males, but males responded better to atomoxetine in terms of impulse control and omission errors (Mei et al. 2022). Estradiol-modulated amphetamine clearance, which is higher in female compared to male rats (Groppetti and Costa 1969), may to some extent explain differential sensitivity to amphetamine. Of note, sex-differences exist in cross-sensitization to methylphenidate and amphetamines Whilst in male rats the cross-sensitization by methylphenidate to amphetamines showed a linear dose–response relationship, in female rats, the intensity of cross-sensitization was U-shaped (Kharas et al. 2019). One plausible explanation for the divergent behavioral outcomes may be sex-specific alterations in neurotransmitter profiles, but the available data are often conflicting and inconclusive.

**Box 3:**
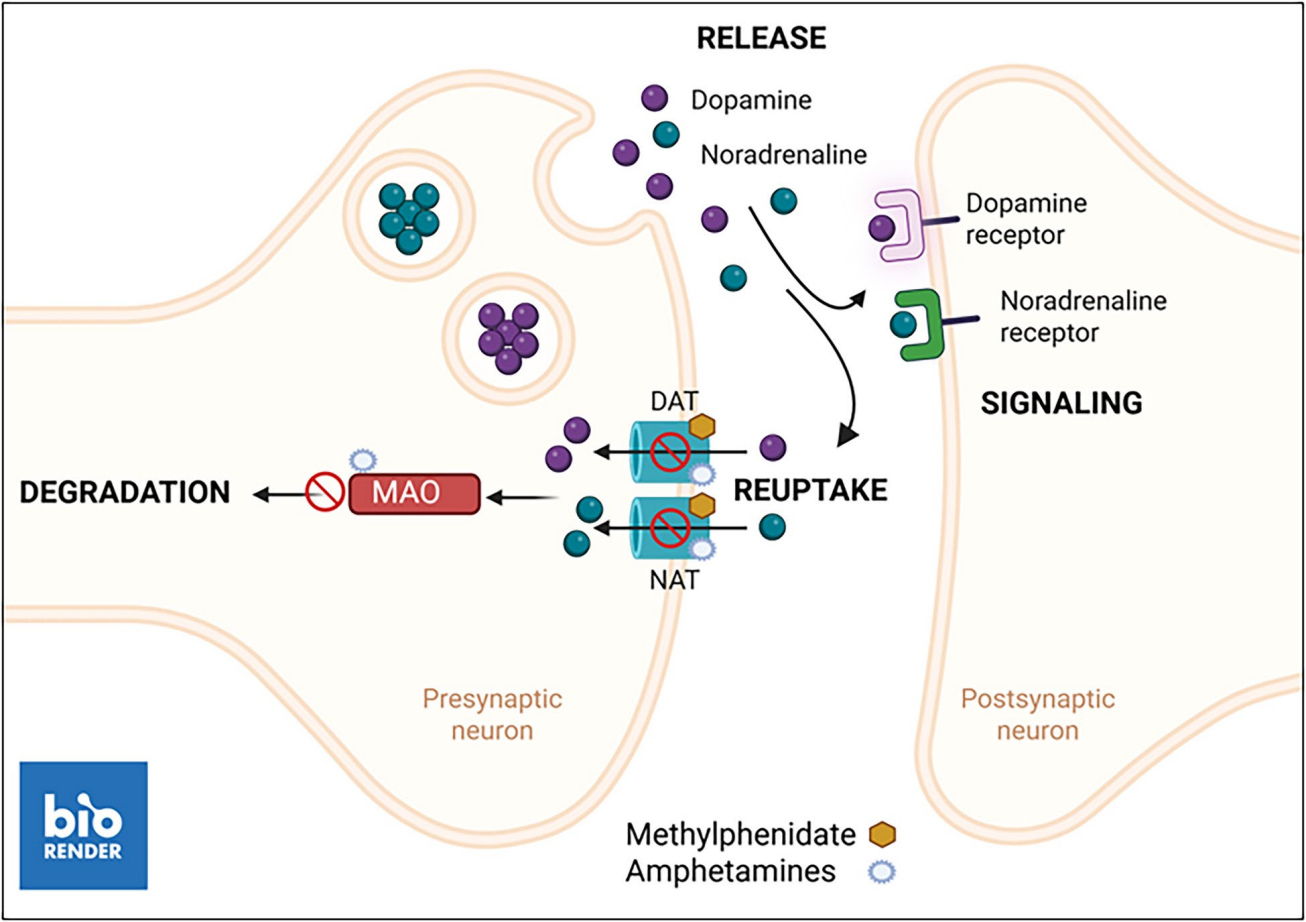
Schematic representation of the primary mechanism of action of methylphenidate and amphetamines in ADHD. Dopamine and noradrenaline are released from presynaptic dopaminergic and noradrenergic neurons. Activation of post-synaptic receptors triggers dopaminergic and noradrenergic signaling, which in ADHD improves executive and attentive function. Methylphenidate and amphetamines block the dopamine and noradrenaline reuptake transporters (DAT, NAT), to sustain neurotransmitter concentrations and receptor activation in the synaptic cleft. Amphetamines additionally block monoamine oxidase (MAO), preventing neurotransmitter degradation. Figure created with Biorender

Amphetamine applied by intraperitoneal injection reduced insular cortical dopamine levels in rats of both sexes but reduced noradrenaline levels only in females (Ruiz et al. 2018). In rat striatal tissue perfused ex vivo, amphetamine-stimulated release of dopamine and noradrenaline was noted in both sexes, but in females was shown to vary with estrous cycle phase. The lowest neurotransmitter release in females was measured during the pro-estrous stage. Ovariectomy of female mice attenuated catecholamine release, with modest restoration of dopamine specifically in response to estradiol of progesterone supplementation. Males castrated with or without testosterone supplementation showed no difference compared to control males (Becker and Ramirez 1981a). In a separate study, the same authors assessed striatal tissues from male and female rats during different stages of development and adulthood. Amphetamine induced release of dopamine at all ages studied regardless of sex. Pre-pubertal castration to induce feminization of male rats, or pre-pubescently androgenized female rats (testosterone propionate or ovariectomy) also retained amphetamine-triggered dopamine release. This response was also retained in male rats castrated in adulthood, by stark contrast, in adult females, ovariectomy led to loss of amphetamine-induced dopamine release within 11 days, sustained for 45 days of observation. The authors suggested that sex-differences in dopamine mobilization under amphetamines may develop during puberty, rather than as a result of neonatal androgen exposure (Becker and Ramirez 1981b).

The close connection between noradrenaline and reward responses is particularly relevant for potential substance abuse facilitated by amphetamine treatment. That the predisposition for multiple substance abuse may be higher in females compared to males is highlighted by preclinical rat studies. Both males and females showed reward enhancement with high-dose amphetamine in combination with nicotine, but in females, a priming of the reward system was evident already at much lower doses of amphetamine with heightened nicotine-evoke reward responses (McNealy et al. 2021). With alcohol the situation may be different however, since repetitive amphetamine dosing during adolescence increased ethanol intake only in adult male rats but not females (Ruiz et al. 2018).

## Implications and considerations for real-life ADHD management

### Growth trajectories and weight effects

The pharmacological treatment of children and adolescents always presents the problem of possible interference in normal development. A recently published analysis of the LIFE (“Leipzig Research Centre for Civilization Diseases”) Child cohort found a potential tendency towards lower *Z* scores for body height and body weight in individuals taking ADHD medication for an extended period compared to the corresponding age- and sex-matched populations (Herzig et al. 2024). Growth trajectory effects of ADHD psychostimulant therapy may be somewhat greater in girls than in boys in terms of height, weight, and body mass index (BMI), but these are overall small and unlikely to pose a significant clinical concern for most children with ADHD treated with stimulants into adolescence (Biederman et al. 2023). In another study, weight changes (typically an initial lowering with later stabilization) were also found to be marginal and not different between the sexes (Al Eid et al. 2024).

### Pharmacovigilance and adverse effects

Overall, children and adolescents with ADHD exposed to ADHD medications do not appear at higher risk of serious cardiovascular events (Houghton et al. 2020; Farhat et al. 2025). However, a comprehensive pharmacovigilance analysis based on the FDA Adverse Event Reporting System (FAERS) recently identified age- and sex- differences in rates and quality of adverse effects under treatment with methylphenidate, amphetamine, and atomoxetine (Wei et al. 2023). Stratified analysis by sex showed no obvious differences in terms of adverse cardiovascular signals in ADHD patients, although QT prolongation on electrocardiogram was reported in adult females treated with methylphenidate, and in atomoxetine-treated males aged 18 years and less. Similarly, growth retardation was noted particularly in atomoxetine-treated males under age of 18, not in females, while the risk of methylphenidate-associated growth disorders was comparable between the sexes, although numerically higher cases numbers occurred in the male cohort. Critically, the risk of suicidal ideation and behavior under methylphenidate, and possibly amphetamines as well, was generally stronger in males. This may arise from differences in impulsivity or drug-induced depression. Alarmingly, a high-risk signal was noted for psychiatric/psychotic disorders and suicidal behaviors in the very young, aged 5 years and less. The data are inadequate to identify a sex-specific component but certainly this aspect of treatment outcome should be closely monitored in children, although its diagnosis remains challenging in the very young.

### Tolerability and drug adherence

Methylphenidate or amphetamines are first-choice agents in ADHD per se, but which of these families and which dosage are best suited for the individual patient is not clear at the outset. Around one quarter of newly referred medication naïve adults with ADHD initiating treatment with stimulants may need to switch from their initially prescribed stimulant family or formulation. The rate of switching has been reported to be significantly higher in those initially prescribed methylphenidate-based therapies compared with those prescribed amphetamines, but in these, there was a greater need to adjust the initially prescribed formulation (Biederman et al. 2021). In this regard, a high baseline emotional dysregulation (ED) appears to contribute to poor amphetamine tolerability particularly in adult males with ADHD.

Adjusting treatment is critical early in therapy, because non-adherence due to intolerance is associated with poorer outcomes (Bijlenga et al. 2017). Particularly female ADHD patients, older patients, those with a higher educational level, and those with a combined ADHD subtype, show higher non-adherence rates. Worse adherence is also noted if treatment is initiated by a primary care provider rather than a psychiatrist (Biederman et al. 2019). For children, additional or other factors may come into play. The Generation R Study in the Netherlands examined child and family characteristics as determinants of methylphenidate adherence and persistence. Better adherence scores were noted if mothers had just one child or an average (rather than high) household income. Higher non-persistence was again identified seen particularly in females and if therapy commenced after the age of 12 (Cheung et al. 2021).

### Sexual behavior

Another outcome that is gaining more attention is the influence of both the underlying ADHD and the superimposed effect of pharmacotherapy on sexual behavior and health. As mentioned earlier, ADHD goes in hand with more frequent risky sexual behavior (Berry et al. 2021; Young et al. 2023), also exemplified by our case presentation of Emilia. A very recent study by Hale et al. (2025) analyzed over 600.000 male and female adolescents with ADHD, classified by use of stimulant, non-stimulant, or no medication. Particularly, the stimulant medications associated with increased libido and hypersexual behaviors, most strongly in males, who also reported higher rates of erectile dysfunction. Stimulant pharmacotherapy in ADHD females was linked with slightly elevated libido and higher rates of contraceptive use. Non-stimulant medications showed fewer sexual side effects overall. The sex-specific impact of ADHD medications, particularly stimulants, on sexual (dys)function and behavior of adolescents requires critical consideration and likely tailored therapeutic management. More personalized treatment is also warranted in the contexts of pregnancy and menopause. Women with ADHD are more prone to postpartum depression after first childbirth and to climacteric mood symptoms (Dorani et al. 2021), how this is affected by prior or ongoing medication remains unclear.

### Quality of life

Impaired health-related quality of life (QoL) is a common clinical feature of ADHD. The quality of life, effectiveness, safety, and tolerability (QU.E.S.T.) study of long-acting amphetamines in adults with ADHD highlighted that symptom improvement was a critical and immediate determinant of mental—but not physical—QoL outcomes. Changes in symptoms were temporally connected to improved QoL, and this interaction was clearly moderated by female sex. The authors further identified patient satisfaction with the medication—which they proposed to reflect the complex interplay of symptom change, tolerability and perception of treatment—to predict self-reported QoL improvement (Weiss et al. 2010). The extent to which ADHD symptoms impact on QoL appears also to be related to the degree of ED, although clear sex differences have not been identified in this interaction (Ben-Dor Cohen et al. 2021). It seems plausible though, that if there were sex-specific improvements of ED in response to pharmacotherapy, this will indirectly lead to sex-specific improvement also in QoL. There is to our knowledge no data on this however. Women may also benefit more strongly from non-pharmacological interventions. The cognitive-functional intervention for adults (Cog-Fun-A), a so-called metacognitive occupation-based intervention for adults with ADHD, led to significant clinical improvements and better QoL scores especially in women, in adherent medication users and in those with better initial cognitive scores, who were more likely to complete the intervention (Buitelaar et al. 2011). A very recently published study highlights the importance of proper ADHD diagnosis, with 3% of a cohort of 50,937 seemingly healthy and undiagnosed individuals (sex match 1:1) displaying ADHD symptoms. Notably, symptom presentation differed between the sexes, with males curiously featuring inattentive symptoms, and females tending to feature hyperactive-impulsive symptoms, which contrasts the typical presentations of diagnosed ADHD. Importantly, the type of symptom presentation modified mental and physical health-related QoL and major depression inventory scores in this undiagnosed cohort (Hald et al. 2025). If and how to initiate pharmacological treatment in this particular clinical context warrants further attention.

### ADHD drug misuse and risk of substance abuse

ADHD is increasingly recognized as a risk factor for alcohol and illicit drug use. In this regard, there may be subtle sex differences in the vulnerability to alcohol and substance abuse, with men possibly more prone than women (Anker et al. 2020), despite the preclinical data (see above) supporting greater susceptibility in females. The data is often conflicting, with some studies suggesting that overall, stimulant therapy for ADHD in adolescents does not significantly increase the risk of drug diversion or later methamphetamine or cocaine misuse, while others provide contrary evidence. In any case, psychotherapy practitioners need to be aware of the possible risks and discuss this with patients and care-givers (Schepis et al. 2020, 2023; Molina et al. 2021; Wong et al. 2022; McCabe et al. 2023; London et al. 2025). Non-prescription methylphenidate use is on the rise amongst young women in particularly, more so than young men. Females more frequently self-medicate to quit opioids, lose weight and treat ADHD-characteristic symptoms, and to a greater degree endorse increased productivity as a benefit of methamphetamine use and accept a number of severe associated risks of methamphetamine (Hooten et al. 2025).

### Off-label use of ADHD medications for other indications

Methylphenidate has been proposed as a candidate approach for weight control in non-ADHD patients, with particularly feasibility for bulimic eating disorders. Acute methylphenidate was shown to suppress appetite, craving and snack-food intake in normal-weight (BMI < 25) and obese (BMI > 30) women, and in normal-weight men, but not obese men. The appetite response to methylphenidate therefore differs between men and women with obesity. This could be relevant for sex-specific weight-loss strategies incorporating dopamine modulation, to which overweight or obese men might be less responsive (Davis et al. 2012, 2016).

## Conclusion

Overall, many gaps in knowledge remain regarding sex differences on all levels of ADHD disease manifestation, diagnosis, and therapy, and the underlying mechanistic basis. Ongoing preclinical work helps to unravel the pathways that determine clinical outcome; although in many cases, this translational data is conflicting. Greater sensitivity and improved study designs will better define the presence and relevance of sex-specific differences in short-term treatment responses and long-term outcomes. Certain treatment strategies are likely to be more or less appropriate, safe and effective in specific patients. How this greater understanding should be implemented in real-life practice warrants further attention in future guideline reforms.

## Data Availability

All source data for this work (or generated in this study) are available upon reasonable request.
